# Light-dependent magnetoreception in birds: the crucial step occurs in the dark

**DOI:** 10.1098/rsif.2015.1010

**Published:** 2016-05

**Authors:** Roswitha Wiltschko, Margaret Ahmad, Christine Nießner, Dennis Gehring, Wolfgang Wiltschko

**Affiliations:** 1 FB Biowissenschaften, Goethe-Universität Frankfurt, Max-von-Laue-Straße 13, 60438 Frankfurt am Main, Germany; 2 Université Pierre et Marie Curie, Casier 156, 4 Place Jussieu, 75005 Paris, France

**Keywords:** avian magnetic compass, cryptochrome 1a, flavin redox cycle, light-activation, radical pairs

## Abstract

The Radical Pair Model proposes that the avian magnetic compass is based on spin-chemical processes: since the ratio between the two spin states singlet and triplet of radical pairs depends on their alignment in the magnetic field, it can provide information on magnetic directions. Cryptochromes, blue light-absorbing flavoproteins, with flavin adenine dinucleotide as chromophore, are suggested as molecules forming the radical pairs underlying magnetoreception. When activated by light, cryptochromes undergo a redox cycle, in the course of which radical pairs are generated during photo-reduction as well as during light-independent re-oxidation. This raised the question as to which radical pair is crucial for mediating magnetic directions. Here, we present the results from behavioural experiments with intermittent light and magnetic field pulses that clearly show that magnetoreception is possible in the dark interval, pointing to the radical pair formed during flavin re-oxidation. This differs from the mechanism considered for cryptochrome signalling the presence of light and rules out most current models of an avian magnetic compass based on the radical pair generated during photo-reduction. Using the radical pair formed during re-oxidation may represent a specific adaptation of the avian magnetic compass.

## 1. Introduction

The Radical Pair Model by Ritz *et al*. [1] proposes that the reception of magnetic directions in birds is based on spin-chemical processes in specialized light-absorbing photopigments, where photon absorption leads to the formation of spin-correlated radical pairs. The magnetic field alters the dynamics of the transition between spin states and thereby modifies the ratio singlet/triplet, with the magnitude of the response depending on the alignment of the radical pair with respect to the direction of the magnetic field. This is assumed to result in an activation pattern across the retina which provides birds with directional information. The model is supported by the observation that radio-frequency fields in the MHz range, a diagnostic tool for radical pair mechanisms [1,2], disrupt magnetic compass orientation of all bird species tested so far [3–8].

As receptor molecule, Ritz *et al*. [1] had suggested cryptochrome, a flavoprotein, where absorption of photons leads to the generation of radical pairs (for details, see e.g. [9]). The avian magnetic compass indeed does not work in the dark [10,11]; it requires short-wavelength light from UV to about 565 nm green (e.g. [12–14]; for summary, see [15]), which is in agreement with an involvement of cryptochrome. A form of this protein, cryptochrome 1a (Cry1a), was found in the UV/V cones in the retina of birds, where it is located at the discs of the outer segment probably in an oriented manner, as required by the Radical Pair Model [16]. Most significantly, activated Cry1a has been detected at the molecular level in the bird retina [17], matching the light conditions that confer magnetic sensitivity [18].

**Figure 1.**
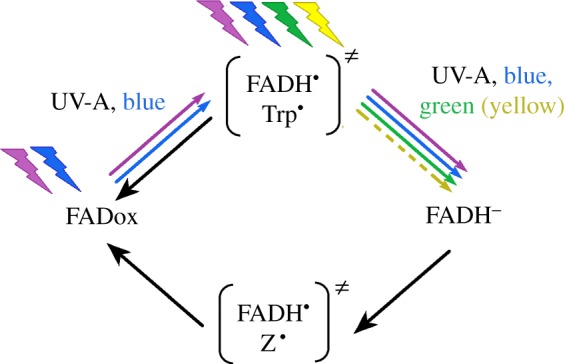
Redox cycle of FAD, the chromophore of cryptochrome. The radical pairs are given in parentheses; coloured arrows, photo-reduction by the respective wavelengths (see text); black arrows, light-independent reactions of re-oxidation (after [19], modified). ‘Z’ in the radical pair generated during re-oxidation stands for a radical whose nature is not yet clear ([21] and text).

The light-absorbing cofactor of cryptochrome is flavin adenine dinucleotide (FAD), which undergoes a redox cycle (e.g. [19,20]). In the dark, it exists in the oxidized, resting form FADox; photo-reduction by UV and blue light up to about 500 nm triggers the transfer of an electron from a nearby tryptophan (Trp), forming the radical pair FADH^•^/Trp^•^. The semiquinone FADH^•^ can be directly re-oxidized in a reaction not requiring light; in the presence of light from UV to about 570 nm green, however, it can be further photo-reduced to the fully reduced form FADH^–^, which is then re-oxidized in a light-independent reaction to FADox. During this re-oxidation, a second radical pair is generated, involving FADH^•^ and a not yet identified radical, possibly O_2_^–•^ (figure 1; for details of the FAD redox cycle, see [19]). This raised the question as to which radical pair is the critical one for detecting magnetic directions.

Given that radical pairs generated during photo-reduction require light, whereas those during flavin re-oxidation are formed independently of light, a simple means of distinguishing between the two possible radical pairs suggested itself: we recorded the orientation of migratory European robins, *Erithacus rubecula*, under flickering light and periodically compensated magnetic field conditions, with the two components arranged in a way that light was on only when the magnetic field was compensated, while the light was off when the local geomagnetic field was present. If the radical pair generated during flavin re-oxidation is the one mediating magnetic directions, then the birds should be able to orient even if the geomagnetic field was present only in the dark.

## 2. Material and methods

The study was performed in January and February 2011, 2012 and 2013 in wooden huts in the Botanical Garden of Frankfurt am Main (50°09′ N, 8°40′ E) where the local geomagnetic field of 47 µT, 66° inclination was largely undisturbed. Testing followed our standard procedure [13,14,18].

### 2.1. Test birds

The test birds were European robins, *Erithacus rubecula* (Turdidae), a night migrating species that is distributed all over Europe. The northern populations migrate and winter in the Mediterranean and northern Africa. Juveniles of probably Scandinavian origin, identified by their wing lengths, were caught in mist-nets during September and October of the previous year in the Botanical Garden and kept over the winter. They were housed in individual cages under a photoperiod that simulated the natural one until the beginning of December, when it was decreased to 8 L : 16 D cycle. Around New Year, the photoperiod was prolonged in two steps to 13 L : 11 D cycle. This induced premature spring migratory activity and allowed us to start the experiments after the first week in January. After the end of the tests, when the photoperiod outside had reached 13 h, the birds were released.

### 2.2. Test apparatus

The robins were tested one at a time in funnel-shaped cages [22] where the inclined walls were lined with thermo-paper. As the birds moved, they left scratches on the paper that documented their activity and could be analysed to calculate their headings.

Each cage was covered with an opaque Plexiglas disc and was placed in a cylinder the top of which was formed by the disc carrying an array of light-emitting diodes (LEDs). We used two narrow band lights, 502 nm turquoise (half-bandwidth 486 and 518 nm), a wavelength where the full redox cycle can run, and 565 nm green light (half-bandwidth 550 and 583 nm) that can induce only the second step of photo-reduction from the semiquinone to the fully reduced form. The light passed through two diffusers before it reached the bird in the cage, where the quantal flux was about 8 × 10^15^ quanta m^−2^ s. The light level was controlled before each test using a radiometer, Optometer P-9710-1 (Gigahertz Optik, Puchheim, Germany) and the probe ‘Visible’ RW-3703-2, a silicon photo element for the wavelength range 400–800 nm, with specific calibrations for the wavelengths of the LEDs used. The light was either steady or was modified with a frequency of 1 Hz. Tests under constant green light in the continuous geomagnetic field served as controls (C G).

Sets of four cages were placed together in the centre of Helmholtz coil systems arranged in a way that their axes coincided with the magnetic vector; when provided with power, the geomagnetic field was compensated about 96% to less than 2 µT, which was controlled by a Fluxgate Magnetometer MAG-01H (Barrington Instruments, Oxford, UK). The coils were also activated in a 1 Hz rhythm.

A custom-made control box controlled the electric current for the lights and the Helmholtz coils. It included a microprocessor with an integrated oscillator operated at 16 MHz (±1%) controlling the timing (Microchip PIC18F26K20). To avoid overshooting electric fields when the Helmholtz coils were powered, the current was slowly increased in small steps within 3.7 ms to reach the voltage required for compensating the geomagnetic field; with this setting, no overshooting electric fields could be measured. Switching the coils off was done correspondingly. In the critical tests where light and geomagnetic field were alternating, the control box was programmed in a way that the coil systems were switched on as described above, and, for a security interval, the current for the light was switched on 15 ms later, which lit the LEDs within 35 ns. When the light was switched off, the LEDs went dark within 105 ns, and the compensation of the magnetic field continued for the respective time of 15 ms. This was to guarantee that there was no overlap between the geomagnetic field and the light. Thus, to avoid an overlap, the duration of the light period and of the period where the geomagnetic field was present were somewhat less than 300 ms and 700 ms, respectively, but we use these numbers for simplicity.

### 2.3. Test performance

The bird room was lit with ‘white’ light including a small portion of near UV. The birds were caught from their housing cages about 10 min before the lights went off in the bird room and brought into the test cages. The test period lasted 1 h. Then the birds were removed from the test cages and returned to their housing cages. The three tests per test condition were performed in ‘rounds’: during round 1, the birds were tested once in each condition (including conditions not included in this study) in a pseudorandom sequence, then in round 2 etc., with the sequence of test conditions differing between rounds.

In order to identify the receptor mechanisms providing the magnetic directions, we also tested the robins with their upper beak anaesthetized by gently rubbing a cotton bud soaked in Xylocaine 2% (Astra Zeneca, Wedel, Germany: active substance: lidocaine hydrochloride). This was to deactivate magnetite-containing structures in the beak. In previous tests, it had cancelled the effect of a strong magnetic pulse [23] and led to disorientation in birds showing ‘fixed direction’ responses (e.g. [10]; for review, see [24]).

### 2.4. Data analysis and statistics

The thermo-paper was removed from the cages and the scratches were counted by a person blind to the type of the test. Tests with less than 35 scratches were discarded because of showing too little activity; these tests were repeated at the end of the series with the same bird in the respective condition. For the activity in 24 sectors, we calculated the heading of the respective test.

The three headings of each bird in each test condition were comprised in a mean vector for that bird with the direction *α*_b_ and the length *r*_b_. Sometimes, birds showed axial behaviour with two of their headings on one end and one on the other end of an axis. In these cases, we calculated the axial vector by doubling the angles and used the preferred end of the axis for further analysis. From the *α*_b_'s of the 12 birds tested in each condition, we calculated the second-order grand mean vector with the direction *α*_N_ and the length *r*_N_. It was tested for significant directional preference using the Rayleigh test [25], and the test conditions were compared with the control using the Mardia Watson Wheeler test for differences in distribution [25]. A median was calculated from the *r*_b_ to characterize the intra-individual variance.

## 3. Results

The results of all tests are presented in table 1, indicating differences to the control tests under steady 565 nm green light, where the birds were oriented in their seasonally appropriate migratory direction. The data of the individual birds are given in the electronic supplementary material, tables S1–S3.

**Table 1.** Orientation in the various test conditions of 12 birds, based on three recordings each. Test conditions: C G, control in continuous 565 nm green light in the geomagnetic field; C T, control in continuous 502 nm turquoise light in the geomagnetic field. G Mag 100/900 and G Mag 300/700, continuous 565 nm green light, geomagnetic field present for 100 ms, compensated for 900 ms and present for 300 ms, compensated for 700 ms, respectively; Li T 300/700, 502 nm turquoise light 300 ms on, 700 ms off, in the geomagnetic field; Li T 300/700Xy, same as before with beak locally anaesthetized with Xylocain; Li G 300/700, 565 nm green light 300 ms on, 700 ms off, in the geomagnetic field; Li T 300/Mag 700, turquoise light on for 300 ms in a compensated magnetic field, geomagnetic field in the dark for 700 ms; Li G 300/Mag 700, green light on for 300 ms in a compensated magnetic field, geomagnetic field in the dark for 700 ms; Li G 300/Mag 700Xy, same as before with the beak locally anaesthetized with Xylocaine. Median *r*_*b*_, median of the 12 birds' vector lengths, reflecting the intra-individual variance; *α_N_*, *r_N_*, direction and length of the grand mean vector (direction in parentheses if not significant), with asterisks indicating a significant directional preference by the Rayleigh test [25]. Δ*C*, angular difference to the mean of the respective control with significance by the Mardia Watson Wheeler test [25] indicated; Δ*X*, angular difference to the difference of the sample above marked X. Significance levels: *p < 0.05, **p < 0.01, ***p < 0.001, n.s., not significant. For the orientation behaviour of individual birds, see the electronic supplementary material, tables S1–S3.

test conditions	year	median *r*_b_	*α_N_*	*r_N_*	Δ*C*	ΔX
C G	2011	0.81	353°	0.70**	C_2011_	
G Mag 100/900	2011	0.80	(148°)	0.08^n.s.^	(+155°)*	
C G	2012	0.94	9°	0.88***	C_2012_	
C T	2012	0.90	8°	0.64**	−1° n.s	
G Mag 300/700	2012	0.89	353°	0.56*	−16° n.s.	
C G	2013	0.96	354°	0.60**	C_2013_	
Li T 300/700	2013	0.84	1°	0.83***	+7° n.s.	X
Li T 300/700 Xy	2013	0.92	5°	0.74***	+11° n.s.	+ 4° n.s.
Li G 300/700	2013	0.95	2°	0.82***	+8° n.s.	
Li T 300/Mag 700	2013	0.95	354°	0.83***	±0° n.s.	
Li G 300/Mag 700	2013	0.97	13°	0.91***	+19° n.s.	X
Li G 300/Mag 700 Xy	2013	0.84	4°	0.81***	+10° n.s.	−9° n.s.

To distinguish a possible effect from unspecific ones, we began with testing whether the birds would be able to orient (i) under flickering light and (ii) when the geomagnetic field was present only part of the time. The respective conditions repeated themselves every second. In this study, we use a behavioural response, with the time birds require for obtaining magnetic directional information for this response yet unknown—it not only depends on the time course of the FAD cycle, but mainly on the biological processes transmitting and processing the respective information, involving, for example, the integration of input from all spatial directions to form a magnetic image [1]. Hence we had to test several timings.

**Figure 2.**
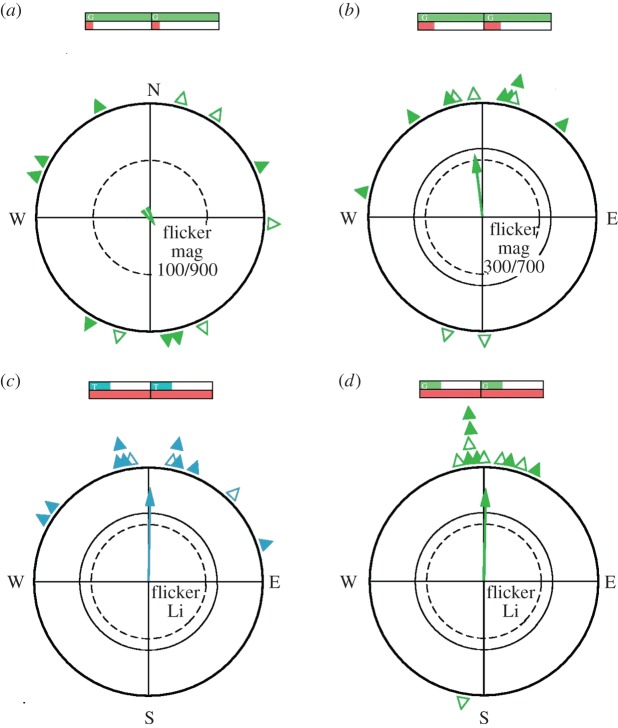
Orientation behaviour during the pre-test series. Above: testing the required duration of the presence of the geomagnetic field under continuous green light, with (*a*) the geomagnetic field present 100 ms s^–1^, 900 ms compensated; (*b*) the geomagnetic field present 300 ms s^–1^, 700 ms compensated. Below: testing flickering light in a constant magnetic field: tests in the geomagnetic field with light present 300 ms s^–1^, 700 ms total darkness. (*c*) Under flickering 502 nm turquoise light; (*d*) under flickering 565 nm green light. The schemes above the circles symbolize the distribution of light (above, green or turquoise) and magnetic field (below; brown). The triangles at the peripheries of the circles mark the mean headings of individual birds based on three recordings each, solid: unimodal, open: preferred end of an axis. The arrows represent the grand mean vectors drawn proportional to the radius of the circle, and the two inner circles mark the 5% (dotted) and the 1% significance border of the Rayleigh test [25].

Under constant green light in a pulsed magnetic field with 100 ms geomagnetic field, 900 ms compensated field (Li G Mag 100/900), the birds were disorientated—obviously, the geomagnetic field present only about 10% of the time each second was too short to allow orientation (table 1 and figure 2*a*). However, the birds were oriented in their seasonally appropriate migratory direction when the geomagnetic field was present 300 ms per second and 700 ms compensated (Li G Mag 300/700) (figure 2*b*). The same was true in the constant geomagnetic field under flickering 502 nm turquoise or 565 nm green light that was 300 ms on and 700 ms off every second (Li T 300/700 Mag and Li G 300/700 Mag; figure 2*c*,*d*). In these cases, the birds' orientation was not different from that in the control tests (table 1), which clearly showed that these periodically changing conditions *per se* did not disrupt orientation by the magnetic compass.

**Figure 3.**
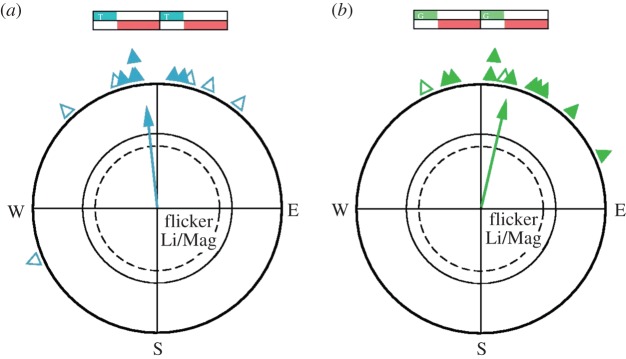
Orientation of birds when the magnetic field and light was present alternatingly. (*a*) Orientation under 502 nm turquoise light when the light was on and the magnetic field compensated for *ca* 300 ms, then the light was off and the geomagnetic field present for *ca* 700 ms, then again the light was on and the magnetic field compensated, etc. (*b*) Orientation in the same condition under 565 nm green light. Symbols as in figure 2.

In the critical tests, the light was on for about 300 ms per second with the magnetic field compensated and the geomagnetic field present only in the dark phase (Li T 300/Mag 700 and Li G 300/Mag 700); we took great care that the light and the geomagnetic field did not overlap. Here, the birds were also oriented in their migratory direction (figure 3), indistinguishable from that in the control condition (table 1).

The orientation under flickering light and when the magnetic field was present only in the dark was not disrupted by local anaesthesia of the upper beak (Li T 300/700 Mag Xy; Li G 300/Mag 700Xy, table 1), indicating that the orienting information in these test conditions still originated in the radical pair processes in the eyes and not in magnetite-based receptors in the beak (see [15,23,24] for discussion).

## 4. Discussion

We discuss our findings in view of the Radical Pair Model with cryptochrome as the molecule forming the radical pairs mediating magnetic directions, because this is the only model that can explain the characteristics of the avian magnetic compass and is supported by experimental evidence (see the electronic supplemental material, S2).

The oriented behaviour of the birds when the magnetic field was present only during the dark intervals shows that the actual detection process of magnetic directions occurs also in the absence of light. If cryptochrome is indeed the receptor molecule, this indicates a crucial role of the radical pair generated during the light-independent re-oxidation of the fully reduced FADH^–^—this radical pair appears to be the one where the modification of the singlet/triplet ratio leads to sensing magnetic directions. It means that for the so-called ‘light-dependent magnetoreception’, in the strict sense, light is not needed for the receptive process itself—this appears to run also in the dark. Light is required, however, to trigger cryptochrome photo-reduction in order to provide the fully reduced form of FADH^–^, because this form is an essential prerequisite for generating, during re-oxidation, the crucial radical pair [19]. The time course for re-oxidation is of the order of minutes [26]; obviously, there is sufficient FADH^–^ available during the dark phase in the geomagnetic field to run the receptive processes before more FADH^–^ is produced during the next light phase.

A similar mechanism had already been suggested by previous experiments [14,15,18,27] when we observed well-oriented behaviour and activated Cry1a under green light, where the first step of photo-reduction cannot run. Yet, this was possible only when birds had been under ‘white’ light before, i.e. under light including UV and blue wavelengths that trigger the photo-reduction of FADox to FADH^•^. Apparently, if at the beginning of the exposure to green light some semiquinone was present, it could be further reduced by green light and then re-oxidized [18,27], forming the magnetically sensitive radical pairs. Our data here provide the first evidence that the radical pair generated during the light-independent re-oxidation is the one underlying the avian magnetic compass.

The new results thus support our previous findings and are in agreement with the magnetic compass of birds being based on Cry1a as receptor molecule. All cryptochromes appear to undergo the same redox cycle [9,19] (figure 1): in the course of photo-reduction and the following light-independent re-oxidation, radical pairs are generated, and these radical pairs react with the magnetic field [1]. In most cases, however, any effect of the ambient (geo)magnetic field is irrelevant for the biological function of the cryptochromes, as they signal the presence and amount of light and entrain the circadian clock [9]. When cryptochromes are controlling biological processes like, for example, hypocotyl growth and flowering in plants, the reactions during photo-reduction of FADox by blue light to the semiquinone FADH^•^ are considered to be crucial (e.g. [20,28,29]). Green light that reduces the amount of FADH^•^ without replenishing it was found to act antagonistically to blue light *in vitro* and also in the living plant [20,29]. This is clearly not the case when magnetic directions are detected by birds [12,14,17,27]: here, blue and green light act synergistically, both causing the further photo-reduction of the semiquinone to the fully reduced FADH^–^, which leads to the formation of the critical radical pair during re-oxidations.

Many theoretical and experimental models of a cryptochrome-based magnetic compass in birds, analogous to the signalling mechanisms of cryptochrome considered in plants, discuss the radical pair FADH^•^/Trp^•^ generated during photo-reduction as being the one mediating magnetic directions (e.g. [30–34]). Our data clearly rule out this possibility. The compatibility of the radical pair FADH^•^/Trp^•^ with magnetoreception is questioned in a recent analysis [35], and theoretical considerations and calculations have indicated that this radical pair formed during photo-reduction may not be optimal for this task because of its magnetic nuclei and hyperfine interactions in both radicals; a radical pair with one radical devoid of magnetic nuclei would be much more effective in responding to the external magnetic field [6,21,36–39], possibly in a ‘reference–probe system’ [39], making use of this difference. The radical pair generated during re-oxidation probably has these properties [6,21] (figure 1). A reference–probe system with FADH^•^ as reference and O_2_^–•^ as probe (e.g. [6,39]), however, raises a number of yet unsolved questions (e.g. [8,38,40,41]). Lee and colleagues [21] suggested that an unknown ‘Z^•^’ radical from the cellular medium could provide an alternative to O_2_^–•^, and the cellular environment can indeed significantly modulate cryptochrome reactions [42]. The involvement of a recently discovered TrpH^•^ radical with a life time of up to 50 µs [35] seems highly unlikely, because the time required for magnetoreception clearly exceeds 100 ms. The specific processes involved in re-oxidation are still poorly understood and require further analysis.

Sensing magnetic directions thus seems to be based on chemical properties that are common to all cryptochromes [9] (figure 1), yet in this case, the radical pair generated during re-oxidation appears to be crucial. A reason for relying on this radical pair could lie in the different nature of the task: instead of signalling the presence of light [9,20,29], cryptochrome has to provide information on directions. In view of the indications that the radical pair generated during re-oxidation could make the better magnetic sensor, it would not be surprising that natural selection had shaped the mechanism in a way that it makes use of the radical pair that is most suitable to solve the required task [6,21]. Together with the location of Cry1a probably in ordered arrays at the discs in the outer segment of the UV/violet cone photoreceptors that are distributed all across the avian retina [16], this could represent a special adaptation to the birds' need for an efficient magnetic compass.

## Supplementary Material

Orientation behaviour of individual birds in the various test conditions

## Supplementary Material

Hypotheses on the mechanisms underlying the avian magnetic compass

## References

[1] Ritz T, Adem S, Schulten K 2000 A model for photoreceptor-based magnetoreception in birds. Biophys. J. 78, 707–718. (10.1016/S0006-3495(00)76629-X) 10653784 PMC1300674

[2] Henbest KB, Kukura P, Rodgers CT, Hore PJ, Timmel CR 2004 Radio frequency magnetic field effects on a radical recombination reaction: a diagnostic test for the radical pair mechanism. J. Am. Chem. Soc. 126, 8102–8103. (10.1021/ja048220q) 15225036

[3] Ritz T, Thalau P, Philllips JB, Wiltschko R, Wiltschko W 2004 Resonance effects indicate a radical-pair mechanism for avian magnetic compass. Nature 429, 177–180. (10.1038/nature02534) 15141211

[4] Wiltschko W, Freire R, Munro U, Ritz T, Rogers L, Thalau P, Wiltschko R 2007 The magnetic compass of domestic chickens, *Gallus gallus*. J. Exp. Biol. 210, 2300–2310. (10.1242/jeb.004853) 17575035

[5] Keary N, Roploh T, Voss J, Thalau P, Wiltschko R, Wiltschko W, Bischof HJ 2009 Oscillating magnetic field disrupts magnetic orientation in zebra finches, *Taeniopygia guttata*. Front. Zool. 6, 25 (10.1186/1742-9994-6-25) 19852792 PMC2774300

[6] Ritz T, Wiltschko R, Hore PJ, Rodgers CT, Stapput K, Thalau P, Timmel CR, Wiltschko W 2009 Magnetic compass of birds is based on a molecule with optimal directional sensitivity. Biophys. J. 96, 3451–3457. (10.1016/j.bpj.2008.11.072) 19383488 PMC2718301

[7] Engels S et al. 2014 Anthropogenic electromagnetic noise disrupts magnetic compass orientation in a migratory bird. Nature 509, 353–356. (10.1038/nature13290) 24805233

[8] Kavokin K, Chernetsov N, Pakomov A, Bojarinova J, Kobylkov D, Namozov B 2014 Magnetic orientation of garden warblers (*Sylvia borin*) under 1.4 MHz radio frequency field. J. R. Soc. Interface 11, 20140451 (10.1098/rsif.2014.0451) 24942848 PMC4208380

[9] Chaves I et al. 2011 The cryptochromes: blue light photoreceptors in plants and animals. Annu. Rev. Plant Biol. 62, 335–364. (10.1146/annurev-arplant-042110-103759) 21526969

[10] Wiltschko W, Wiltschko R 1981 Disorientation of inexperienced young pigeons after transportation in total darkness. Nature 291, 433–434. (10.1038/291433a0)

[11] Stapput K, Thalau P, Wiltschko R, Wiltschko W 2008 Orientation of birds in total darkness. Curr. Biol. 18, 602–606. (10.1016/j.cub.2008.03.046) 18424144

[12] Wiltschko W, Munro U, Ford H, Wiltschko R 1993 Red light disrupts magnetic orientation of migratory birds. Nature 364, 525–527. (10.1038/364525a0)

[13] Wiltschko W, Wiltschko R 1995 Migratory orientation of European robins is affected by the wavelength of light as well as by a magnetic pulse. J. Comp. Physiol. A 177, 363–369. (10.1007/BF00192425)

[14] Wiltschko W, Wiltschko R 1999 The effect of yellow and blue light on magnetic compass orientation in European robins, *Erithacus rubecula*. J. Comp. Physiol. A 184, 295–299. (10.1007/s003590050327)

[15] Wiltschko R, Stapput K, Thalau P, Wiltschko W 2010 Directional orientation of birds by the magnetic field under different light conditions. J. R. Soc. Interface 7(Suppl 2), S163–S178. (10.1098/rsif.2009.0367.focus) 19864263 PMC2843996

[16] Nießner C, Denzau S, Gross JC, Peichl L, Bischof HJ, Fleissner G, Wiltschko W, Wiltschko R 2011 Avian ultraviolet/violet cones identified as probable magnetoreceptors. PLoS ONE 6, 20091 (10.1371/journal.pone.0020091) PMC3102070 21647441

[17] Nießner C, Denzau S, Stapput K, Ahmad M, Peichl L, Wiltshcko W, Wiltschko R 2013 Magnetoreception: activated cryptochrome 1a concurs with magnetic orientation in birds. J. R. Soc. Interface 10, 20130618 (10.1098/rsif.2013.0638) PMC3785833 23966619

[18] Wiltschko R, Gehring D, Denzau S, Nießner C, Wiltschko W 2014 Magnetoreception in birds: II. Behavioural experiments concerning the cryptochrome cycle. J. Exp. Biol. 217, 4225–4228. (10.1242/jeb.110981) 25472973 PMC4254397

[19] Müller P, Ahmad M 2011 Light-activated cryptochrome reacts with molecular oxygen to form a flavin-superoxide radical pair consistent with magnetoreception. J. Biol. Chem. 286, 21 033–21 040. (10.1074/jbc.M111.228940) PMC3122164 21467031

[20] Banerjee R, Schleicher E, Meier S, Moñoz Viana R, Pokorny R, Ahmad M, Bittl R, Batschauer A 2007 The signaling state of *Arabidopsis* cryptochrome 2 contains flavin semiquinone. J. Biol. Chem. 282, 14 916–14 922. (10.1074/jbc.M700616200) 17355959

[21] Lee AA, Jason CS, Hogben HJ, Biskup T, Kattnig DB, Hore PJ 2013 Alternative radical pairs for cryptochrome-based magnetoreception. J. R. Soc. Interface 11, 20131063 (10.1098/rsif.2013.1063) PMC4006233 24671932

[22] Emlen ST, Emlen JT 1966 A technique for recording migratory orientation of captive birds. Auk 83, 361–367. (10.2307/4083048)

[23] Wiltschko W, Munro U, Ford H, Wiltschko R 2009 Avian orientation: the pulse effect is mediated by the magnetite receptors in the upper beak. Proc. R. Soc. B 276, 2227–2232. (10.1098/rspb.2009.0050) PMC2677601 19324756

[24] Wiltschko R, Wiltschko W 2013 The magnetite-based receptors in the beak of birds and their role in avian navigation. J. Comp. Physiol. A 199, 89–98. (10.1007/s00359-012-0769-3) PMC3552369 23111859

[25] Batschelet E 1981 Circular statistics in biology. New York, NY: Academic Press.

[26] Herbel V, Orth C, Wenzel R, Ahmad M, Bittl R, Batschauer A 2013 Lifetimes of *Arabidopsis* cryptochrome signaling states *in vivo*. Plant J. 74, 583–592. (10.1111/tpj.12144) 23398192

[27] Nießner C, Denzau S, Peichl L, Wiltschko W, Wiltschko R 2014 Magnetoreception in birds: I. Immunohistochemical studies concerning the cryptochrome cycle. J. Exp. Biol. 217, 4221–4224. (10.1242/jeb.110965) 25472972 PMC4254396

[28] Berndt A, Kottke T, Breitkreuz H, Dvorsky R, Henning S, Alexander M, Wolf E 2007 A novel photoreaction mechanism for the circadian blue light photoreceptor *Drosophila* cryptochrome. J. Biol. Chem. 282, 13 011–13 021. (10.1074/jbc.M608872200) 17298948

[29] Bouly JP et al. 2007 Cryptochrome blue light photoreceptors are activated through interconversion of flavin redox states. J. Biol. Chem. 282, 9383–9391. (10.1074/jbc.M609842200) 17237227

[30] Izmaylov AF, Tully JC, Frisch MJ 2009 Relativistic interactions in the radical pair model of magnetic field sense in CRY-1 protein of *Arabidopsis thaliana*. J. Phys. Chem. A 113, 12 276–12 284. (10.1021/jp900357f) 19863135

[31] Weber S et al. 2010 Origin of light-induced spin-correlated radical pairs in cryptochrome. J. Phys. Chem. 114, 14 745–14 754. (10.1021/jp103401u) PMC4329313 20684534

[32] Maeda K et al. 2012 Magnetically sensitive light-induced reactions in cryptochrome are consistent with its proposed role as a magnetoreceptor. Proc. Natl Acad. Sci USA 109, 4774–4779. (10.1073/pnas.1118959109) 22421133 PMC3323948

[33] Solov'yov IA, Domratcheva T, Moughal Shahi AR, Schulten K 2012 Decrypting cryptochrome: revealing the molecular identity of the photoactivation reaction. J. Am. Chem. Soc. 134, 18 046–18 052. (10.1021/ja3074819) PMC3500783 23009093

[34] Solov'yov A, Schulten K 2012 Reaction kinetics and mechanism of magnetic field effects in cryptochrome. J. Phys. Chem. B 116, 1089–1099. (10.1021/jp209508y) 22171949 PMC3266978

[35] Müller P, Yamamoto J, Martin R, Iwai S, Brettel K 2015 Discovery and functional analysis of a 4th electron-transferring tryptophan conserved exclusively in animal cryptochromes and (6-4) photolyases. Chem. Commun. 51, 15 502–15 505. (10.1039/C5CC06276D) 26355419

[36] Rodgers CT, Norman SA, Henbest KB, Timmel CR, Hore JP 2007 Determination of radical re-encounter probability distributions from magnetic field effects. J. Am. Chem Soc. 129, 6746–6755. (10.1021/ja068209l) 17469816

[37] Maeda K, Henbest KB, Cintolesi F, Kuprov I, Rodgers CT, Liddell PA, Gust D, Timmel CR, Hore JP 2008 Chemical compass model of avian magnetoreception. Nature 453, 387–390. (10.1038/nature06834) 18449197

[38] Solov'yov IA, Schulten K 2009 Magnetoreception through cryptochrome may involve superoxide. Biophys. J. 96, 4804–4813. (10.1016/j.bpj.2009.03.048) 19527640 PMC2712043

[39] Ritz T, Ahmad M, Mouritsen H, Wiltschko R, Wiltschko W 2010 Photoreceptor-based magnetoreception: optimal design of receptor molecules, cells, and neuronal processing. J. R. Soc. *Interface* 7(Suppl 2), S135–S146. (10.1098/rsif.2009.0456.focus) PMC2843994 20129953

[40] Hogben HJ, Efimova O, Wagner-Rundell N, Timmel CR, Hore PJ 2009 Possible involvement of superoxide and dioxygen with cryptochrome in avian magnetoreception: origin of Zeemann resonances observed by *in vivo* EPR spectroscopy. Chem. Phys. Lett. 480, 228–233. (10.1016/j.cplett.2009.08.051)

[41] Kavokin KV 2009 The puzzle of magnetic resonance effect on the magnetic compass of migratory birds. Bioelectromagnetics 30, 401–410. (10.1002/bem.20485) 19291711

[42] Engelhard C, Wang X, Robles D, Moldt J, Essen LO, Batschauer A, Bittl R, Ahmad M 2014 Cellular metabolites enhance the light sensitivity of *Arabidopsis* cryptochrome through alternate electron transfer pathways. Plant Cell 26, 4519–4531. (10.1105/tpc.114.129809) 25428980 PMC4277212

