# Supplementary material for: Light-dependent magnetoreception in birds: the crucial step occurs in the dark
Source: J R Soc Interface. 2016 May;13(118):20151010. doi: 10.1098/rsif.2015.1010 (PMC4892254; doi:10.1098/rsif.2015.1010)
Supplement: Orientation behaviour of individual birds in the various test conditions [file rsif20151010supp1.pdf]

SUPPLEMENTARY MATERIAL 1

Light-dependent magnetoreception in birds: The crucial step occurs in the dark

Roswitha Wiltschko, Margaret Ahmad, Christine Nießner, Dennis Gehring and Wolfgang Wiltschko

1. Orientation behaviour of individual birds in the various test conditions

n, number of evaluable tests per birds; α_b, $r_{b,}$ direction and length of the birds' mean vectors, with ^{A} indicating axial vectors where the preferred end of the axis is indicated.

Test conditions:

Control: under constant 565 nm green light in the continuous geomagnetic field

Control T: under constant 502 nm turquoise light in the continuous geomagnetic field

Li G Mag 100/900: under constant green light, geomagnetic field present 100 ms per second

Li G Mag 300/700: under constant green light, geomagnetic field present 300 ms per second

Table S1: Spring 2011

	Control			Li G Mag 100/900		
Bird	n	α_b	r_b	n	α_b	r_b
10-2	3	4°	0.66	3	195°	1.00^A
10-7	3	342°	0.58	3	96°	0.61^A
10-18	3	240°	0.45^A	3	65°	0.89
10-29	3	184°	0.62^A	3	290°	0.63
10-20	3	348°	0.97	3	213°	0.87
10-21	3	355°	0.84	3	47°	0.53^A
10-23	3	11°	0.77^A	3	298°	0.95
10-24	3	358°	0.93	3	307°	0.95
10-25	3	6°	0.75	3	93°	0.59^A
10-26	3	360°	0.94	3	154°	0.08^A
10-27	3	3°	0.92	3	172°	0.94
10-28	3	6°	0.93	3	15°	0.78^A

Table S2: Spring 2012

	Control			Li G Mag 300/700			Control T		
Bird	n	α_b	r_b	n	α_b	r_b	n	α_b	r_b
11-27	3	351°	0.93^A	3	10°	1.00	3	343°	0.08^A
11-14	3	11°	0.98	3	13°	0.98	3	26°	0.93
11-15	3	77°	0.60	3	344°	0.94	3	357°	0.90
11-16	3	348°	0.94^A	3	15°	0.84^A	3	191°	0.90^A
11-17	3	30°	0.85	3	197°	0.92^A	3	348°	0.48^A
11-18	3	12°	0.98	3	15°	0.96	3	18°	0.95^A
11-19	3	11°	0.99^A	3	179°	0.44^A	3	355°	0.76^A
11-20	3	310°	0.53	3	347°	0.58^A	3	5°	0.98^A
11-21	3	19°	0.98	3	325°	0.86	3	32°	0.91
11-22	3	350°	0.79^A	3	282°	0.49	3	186°	0.74^A
11-23	3	3°	0.97	3	41°	0.94	3	15°	0.65
11-24	3	27°	0.91	3	356°	0.76^A	3	19°	0.95

Test conditions:

Li T 300/700 Mag: under flickering 502 nm turquoise light, on 300 ms per second, in the constant geomagnetic field

Li T 300/700 Mag Xy: as above, but upper beak anesthetised with Xylocaine

Li G 300/700 Mag: under flickering 565 nm green light, on 300 ms per second, off 700 ms, in the constant geomagnetic field

Li T 300 /Mag 700: 300 ms per second turquoise light in a compensated magnetic field; the remaining ca. 700 ms in the geomagnetic field in the dark

Li G 300 /Mag 700: 300 ms per second green light in a compensated magnetic field; the remaining ca. 700 ms in the geomagnetic field in the dark

Li G 300 G/Mag 700 Xy: as above, but upper beak anesthetised with Xylocaine

Table S3: Spring 2013

	Control			Li T 300/700 Mag			Li T 300/700 Mag Xy			Li G 300/700 Mag		
Bird	n	α_b	r_b	n	α^b	r_b	n	α_b	r_b	n	α_b	r_b
12-13	3	202°	0.39 [A]	3	11°	0.68[A]	3	190°	0.97[A]	3	355°	0.99
12-14	3	332°	0.95	3	301°	0.56	3	11°	0.90	3	355°	0.96
12-15	3	32°	0.98 [A]	3	352°	0.84[A]	3	342°	1.00	3	8°	0.92[A]
12-17	1	3°	(1.00)	1	72°	(1.00)	1	7°	(1.00)	2	12°	0.95
12-18	3	9°	0.93	3	14°	0.96	3	347°	0.99	3	348°	0.94[A]
12-24	3	293°	0.81	3	12°	1.00	3	307°	0.92	3	25°	1.00
12-30	3	205°	0.93 [A]	3	22°	0.99	3	358°	0.58[A]	3	358°	0.97
12-31	3	1°	0.99	3	349°	0.82	3	24°	0.98	3	360°	0.83[A]
12-32	3	4°	1.00	3	308°	0.84	3	8°	0.84[A]	3	18°	0.80[A]
12-33	3	16°	0.96 [A]	3	346°	0.98	3	53°	0.54	3	190°	0.69[A]
12-34	3	16°	0.99	3	44°	0.52[A]	3	6°	0.97	3	353°	0.97
12-35	3	358°	0.98	3	348°	0.97	3	33°	0.92[A]	3	354°	0.64[A]

Table S3, continued: Spring 2013

	Li T 300 /Mag 700			Li G 300 /Mag 700			Li G 300 G/Mag 700 Xy		
Bird	n	α_b	r_b	n	α_b	r_b	n	α_b	r_b
12-13	3	23°	0.59[A]	3	25°	0.99	3	20°	0.60[A]
12-14	3	345°	1.00	3	4°	0.97	3	18°	0.98
12-15	3	11°	0.91	3	10°	0.25[A]	3	19°	0.96
12-17	2	351°	1.00	2	335°	0.96[A]	1	30°	(1.00)
12-18	3	350°	0.98	3	43°	0.80	3	3°	0.83
12-24	3	7°	0.99	3	345°	0.93	3	5°	0.71[A]
12-30	3	342°	0.99[A]	3	348°	0.97	3	6°	0.98
12-31	3	14°	0.88[A]	3	13°	0.97	3	197°	0.74[A]
12-32	3	247°	0.90[A]	3	5°	0.99	3	349°	0.84
12-33	3	38°	1.00[A]	3	65°	0.75	3	351°	0.86
12-34	3	351°	0.80	3	23°	0.99	3	343°	0.95
12-35	3	318°	0.65[A]	3	28°	0.91	3	352°	0.41[A]

Bird 12-17 escaped during the experiments
