# Supplementary material for: Light-dependent magnetoreception in birds: the crucial step occurs in the dark
Source: J R Soc Interface. 2016 May;13(118):20151010. doi: 10.1098/rsif.2015.1010 (PMC4892254; doi:10.1098/rsif.2015.1010)
Supplement: Hypotheses on the mechanisms underlying the avian magnetic compass [file rsif20151010supp2.pdf]

SUPPLEMENTARY MATERIAL 2

Light-dependent magnetoreception in birds: The crucial step occurs in the dark

Roswitha Wiltschko, Margaret Ahmad, Christine Nießner, Dennis Gehring and Wolfgang Wiltschko

2. Hypotheses on the mechanisms underlying the avian magnetic compass

Here, we give a brief summary of the hypotheses on the reception mechanisms providing birds with directional information from the magnetic field and their status concerning experimental support.

The avian magnetic compass has three typical characteristics: (1) it is an inclination compass not responding to the polarity of the magnetic field, but using the axis of the field lines and their inclination in space, (2) it has a fairly narrow biological window around the intensity of the ambient field, which is flexible, being able to adjust to other intensities, and (3) it requires short-wavelength light from UV to about 565 nm green (see [S1]).

The Radical Pair Model forwarded by Ritz and colleagues assuming that magnetic directional information is mediated by a radical pair mechanism, with cryptochrome, a photo-pigment with flavin adenine dinucleotide (FAD) as chromophore forming the radical pairs, is the only model so far that can explain these characteristics. The model with the experimental and histological evidence supporting it (references [1-18] in the main text) is briefly described in the Introduction of the paper. So far, all findings on magnetic compass orientation in birds are in agreement with this model.

Other mechanisms of magnetoreception, mainly based on magnetite, a ferromagnetic iron oxid, have been suggested, but there are conflicting findings. Superparamagnetic and single domain magnetite was described in the skin of the upper beak and the ethmoid region of birds (e.g.[S2-S6], but see [S7]); these receptors mediate magnetic information through the trigeminal nerve [S3;S8,S9]. However, this information does not seem to be part of the avian magnetic compass, because anaesthesia of the upper beak or disrupting the trigeminal nerve does not affect the compass orientation of migratory birds [S10-S12]). The latter also clearly speaks against a hypothesis suggesting integration of magnetite-based information and radial pair processes in providing compass information [S13]. Instead, the magnetite-based receptors seem to mediate information of magnetic intensity for navigation, as de-activating them or disrupting the trigeminal nerve suppresses navigational responses in migrants [S14] and removes the effect of a strong magnetic anomaly on the initial orientation of displaced homing pigeons [S15]. However, under certain light regimes when the avian magnetic compass is disrupted, migratory birds show so-called ‘fixed direction responses’, a behaviour that is different from the migratory orientation and does not change between spring and autumn. It seems to originate in the magnetite-based receptors, for anaesthetising the beak suppresses this behaviour, leading to disorientation [S16,S17]. For a review on these receptors, see [S18].

In 2011, Wu and Dickman reported electrophysiological responses in higher centres of the avian brain, which they assumed to originate in the lagena, part of the inner ear [S19]. Iron-rich particles were indeed found in the hair cells of the inner ear, but they are ferritin corpuscles not suitable for mediating magnetic information [S20,S21].- Information from the inner ear may be involved in the avian magnetic compass, but this is most likely information on gravity: as the radical pair processes are not sensitive for the polarity of the magnetic field; in order to distinguish between the two ends of the axis of the field lines, birds have to consider the inclination of the field lines, probably with the help of gravity [S22].

References:

S1 Wiltschko R, Wiltschko W. 2014 Sensing magnetic directions in birds: radical pair processes involving cryptochrome .*Biosensors* **4**, 221-242.

S2 Beason RT, Brennan WJ. 1986 Natural and induced magnetization in the bobolink, *Dolichonyx orizivorus* (Aves: Icteridae). *J. Exp. Biol.* **125**, 49-56.

S3 Willams MN, Wils JM. 2001 Trgeminally innervated irnin-containign structures in the beak of homing pigeons, and other birds. Brain. Res. **889**, 243-246.

S4 Fleissner G, Holtcamp-Rötzler E, Hanzlik M, Winkhofer M, Fleissner G, Petersen N, Wiltschko W. 2003 Ultrastructural analysis of a putative magnetoreceptor in the ebak of homing pigeons. *J. Comp. Neurol.* **458**, 350-360.

S5 Tian L, Xiao B, Lin W, Zhang S, Zhu R, Pan Y. 2008 Testing for the presence of magnetite in the upper-beak skin of homing pigeons. *Biometals* **20**, 197-204.

S6 Falkenberg G, Fleissner G, Schuchardt K, Kuehbacher M, Thalau P,Mouritsen H, Heyers D, Wellenreuther G, Fleissner G. 2010 Avian magnetoreception: elaborate iron mineral containing dentrites in the upper beak seem to be a common feature of birds. *PLoS ONE 5*, e9231.

S7 Treiber CD, Salzer MC, Riegler J, Edelmann N, Sugar C, Breuss M, Pichler P, Cardiou H, Saunders M, Shaw J, Keays DA 2012 Clusters of iron-rich cells in the upper beak of pigeons are macrophages not magnetosensitive neurons. *Nature 484*, 367-370.

S8 Semm P., Beason RC. 1990 Responses to small magnetic variations by the trigeminal system of the bobolink. *Brain. Res. Bull.* **25**, 735-740.

S9 Heyers D, Zapka M, Hoffmeister M, Wild JM, Mouritsen H. 2010 Magnetic field changes activae the trigeminal brainstem complex in a migratory bird. *Proc. Natl. Acad. Sci. USA* **107**, 9394-9399.

S10 Beaon RC, Semm P. 1996 Does the avian ophthalmic nerve catty magnetic information? *J. Exp. Biol.* **199**, 1241-1244.

S11 Wiltschko W, Munro U, Ford H, Wiltschko R. 2009 Avian orientation: the pulse effect is mediated by the magnetite receptors in the upper beak. *Proc. R. Soc.* **B** 276, 2227-2232.

S12 Zapka M, Heyers D, Hein CM, Engels S., Schneidr, NL, Hans J, Weiler S, Dreyer D, Kihskinev D, Wild JM, Mouritsen H. 2009 Visual but not trigeminal mediationof magnetic compass information in a migratory bird. *Nature* **461**, 1274-1278.

S13 Kirschvink JL, Winklhofer M, Walker MM. Biophysics of magnetic orientation: strengthening the interface between theory and experimental design. *J. R. Soc. Interface* **7**, *Suppl.* **2**, S179-S191.

S14 Kishkinew D, Chernetsov N, Mouritsen H. 2013 Migratory reed warblers need intact trigeminal nerves to correct for a 1,000 km eastward displacement. *PLoSONE* **8**, e65847.

S15 Wiltschko R, Schiffner I, Fuhrmann P, Wiltschko W. 2010. The role of the magnetite-based receptors in the beak in pigeon homing. *Curr. Biol.* **20**, 1534-1538.

S16 Stapput K, Thalau P, Wiltschko R, Wiltschko W. 2008 Orientation of birds in total darkness. *Curr. Biol.* **18**, 603-806.

S17 Wiltschko R, Dehe L, Gehring D, Thalau P, Wiltschko W. 2013 Interactions between the visual and the magnetoreception system: Different effects of bichromatic light regimes in the directional behavior of migratory birds.*J. Physiol. Paris* **107**, 137-146.

S18 Wiltschko R, Wiltschko W. 2013 The magnetite-based receptors in the beak of birds and their role in avian navigation. *J. Comp. Physiol. A* **199**, 89-98.

S19 Wu LO, Dickman JD. 2011 Magnetoreception in an avian brain in part mediated by inner ear lagena. *Curr. Biol.* **21**, 418-423.

S20 Lauwers M, Pichler P, Edelman NB, Resch GP, Ushakova L, Salzer MC, Heyers D, Daunders M, Shaw J, Keays DA 2013 An iron-rich organelle in the cuticular plate of avian hair cells. *Curr. Biol.* **23**, 924-929.

S21 Jandacka P, Burda H, Pistora J. 2014 Magnetically induced behavior of ferritin corpuscles in avian ears: can cuticulosomes function as magnetosomes? *J. R. Soc. Interface* **12**, 20141087

S22 Wiltschko W, Wiltschko R. The magnetic compass of European robins. *Science* **176**, 62-64.
